# A Radioresponse-Related lncRNA Biomarker Signature for Risk Classification and Prognosis Prediction in Non-Small-Cell Lung Cancer

**DOI:** 10.1155/2021/4338838

**Published:** 2021-09-21

**Authors:** Jiahang Song, Shuming Zhang, Yuanyuan Sun, Junjie Gu, Ziqi Ye, Xinchen Sun, Qiyun Tang

**Affiliations:** ^1^Department of Gerontology, The First Affiliated Hospital of Nanjing Medical University, Nanjing 210000, China; ^2^Department of Radiation Oncology, The First Affiliated Hospital of Nanjing Medical University, Nanjing 210000, China

## Abstract

**Purpose:**

Radiotherapy resistance is now recognized as the major obstacle to the effective therapeutic management of non-small-cell lung cancer (NSCLC). As a single biomarker has limited effect in stratifying NSCLC patients, this research aimed to identify long non-coding RNAs (lncRNAs) correlated with radiotherapy response to ameliorate forecast of NSCLC prognosis.

**Methods:**

In a cohort of NSCLC patients with radiotherapy history (*n* = 96) from TCGA, genetic data of lncRNA expression profiling were performed. To identify radioresponse-related lncRNA sets which dysregulated significantly between radiosensitive (RS) and radioresistant (RR) groups, differential expression analysis was carried out. Cox relative regression was implemented to set up a radioresponse-related risk model. Moreover, we adopted survival analysis to measure the predictive potentiality of the prognosis model.

**Results:**

Four radioresponse-related lncRNAs (CASC19, LINC01977, LINC02471, and MAGI2-AS3) were screened to create a prognostic signature. Then, we described a lncRNA signature-based regulatory network and explored the correlation of the immune microenvironment and the signature. Additionally, *in vitro* assays uncovered inhibition of LINC01977 weakened radioresistance of NSCLC cells.

**Conclusion:**

We provided a novel radioresponse-related lncRNAs signature with excellent clinical potency for an effective prognostic forecast of patients.

## 1. Introduction

Lung cancer is the most prevalent thoracic tumor with the second incidence of malignancy in the world [1]. Non-small-cell lung cancer (NSCLC) makes up nearly 85% of lung cancers, whereas 50% to 75% of patients are already diagnosed at the late stages, losing the opportunity for early surgical resection [2]. Therefore, radiotherapy (RT) has increasingly become the main clinical treatment choice. In fact, radiotherapy, as an active and effective therapeutic modality, has played a central part in the whole process management of lung cancer [3]. Nonetheless, the persistence of local control of NSCLC by radiotherapy still has the problems of inevitable recurrence and tolerance [4]. It has been reported that the incidence of local failure of radiotherapy in patients with lung cancer is 24%–40%. Even with concurrent radical radiotherapy and chemotherapy, there is a local failure rate of up to 30%–50% [5]. In conclusion, radiation resistance will still limit the long-term control of lung cancer and eventually lead to local control failure and disease progression of lung cancer [6]. Consequently, we need to further explore the molecular mechanism and develop novel biomarkers, so as to improve the response rate of radiotherapy and overcome resistance to radiotherapy in NSCLC.

lncRNAs belong to the noncoding RNA family with a length of over 200 nucleotides, which usually are not capable of encoding proteins [7]. With the gradual in-depth study of lncRNA, they have been shown to participate in a variety of tumor initiation and development [8–10]. Currently, a total of 7,942 cancer-related lncRNAs were identified to be biomarkers for specific particular tumor types by Iyer and colleagues [11]. Accordingly, better comprehension of the effects of lncRNAs in tumors can favor the development of new diagnostic markers and the exploitation of prospective therapeutic targets. Increasing evidence has explicitly pointed out that radioresistance-related lncRNAs also play crucial roles in the regulation of tumor radiation sensitivity. For instance, lncRNA KCNQ1OT1 can interact with miR-372-3p to enhance stereotactic body radioresistance by inducing autophagy in lung adenocarcinoma [12]. HOX transcript antisense RNA (HOTAIR), best known for regulating transcription, contributes to tumor radioresistance via miR-93/ATG12 [13]. However, prognostic biomarkers based on expression profiles of radioresistance-related lncRNA have not been elaborated in NSCLC.

This article aimed to set up a radioresistance-related lncRNA prognostic model with an integrated bioinformatics analysis, which predicts the prognosis of patients with NSCLC undergoing RT. First, the lncRNAs which correlated with RT response were collected from 96 TCGA-NSCLC cases with lncRNA expression profiles. Then, we built up a radioresistance-related signature consisting of four lncRNAs and verified its accuracy and reliability in different cohorts. In addition, the relationship between immunity and signature was compared by combining the ssGSEA method and immunity-associated algorithm. Finally, we confirmed that these four lncRNAs were markedly dysregulated in NSCLC cell lines by a qRT-PCR assay. Moreover, we observed that the downregulation of LINC01977 significantly blocked the clonogenic survival of NSCLC cells after irradiation.

## 2. Methods

### 2.1. Data Source and Processing

The TCGA-NSCLC lncRNA-seq expression profiles and related clinical data were collected from the TCGA project (https:/portal.gdc.cancer.gov/). A total of 96 NSCLC patients who received RT were selected for this study. Our inclusion criteria for patients were as follows: (1) histologically diagnosed with NSCLC; (2) available expression profiles; and (3) overall survival time greater than 30 days. We divided 96 cases into the radiosensitive (RS) group (*n* = 53) and radioresistant (RR) group (*n* = 43) based on the radiotherapy response. Patients who presented complete remission after RT were recognized as radiosensitive whereas those showing stable disease and progressive disease after RT were considered radioresistant. All patients' clinical characteristics are shown in Table 1.

In order to obtain radioresponse-related lncRNAs (RRlncRNAs) in NSCLC, we analyzed the differential expression of lncRNAs between the RS group and RR group by limma in R software (|fold change (FC)| = 1.0 and *p* < 0.05) [14].

### 2.2. Identification of RRlncRNAs Risk Score Model

To get an optimal RRlncRNAs model, we randomly divided 96 patients into the training set and test set at 1 : 1 ratio. In the training set, candidate prognostic lncRNAs were firstly obtained by the univariate hazard Cox method based on RRlncRNAs. Next, least absolute shrinkage and selection operator (LASSO) penalized analysis with multivariate regression method were carried to set up a novel RRlncRNAs signature through the package “glmnet.” We utilized the following formula to generate the risk score of NSCLC patients: risk score = (exp RRlncRNA 1 × coef) + (exp RRlncRNA 2 × coef) + … + (exp RRlncRNA *n* × coef). The exp means the expression value of each RRlncRNA and the coef is the coefficient of each RRlncRNA generated by Cox relative analysis. According to the median value of risk score, all cases with RT history were split into high- and low-risk score groups, and the differences in survival outcome were compared using K-M analysis.

### 2.3. Establishment and Validation of Nomogram

The data about 96 patients with complete clinical information was integrated with the risk signature, and the independent prognostic analysis was conducted by the “survival” package [15]. Furthermore, the RT response-based nomogram was established according to risk scores and other clinicopathological factors to forecast the prognosis of patients. Subsequently, the accuracy of the prognostic nomogram was verified according to the calibration curves which were generated by comparing the nomogram prediction ability and the observation for the 1-year, 3-year, and 5-year OS rates.

### 2.4. Gene Set Enrichment Analysis (GSEA)

We employed GSEA to distinguish the important functional phenotypes between the high-risk group and the low-risk group. After 1000 substitutions, an enriched gene set was obtained based on a nominal *p* value <0.05. The Hallmark (v7.4) gene sets were downloaded from the Molecular Signatures Database v7.4 download page (https://www.gsea-msigdb.org/gsea/downloads.jsp). GSEA was conducted based on the downloaded gene sets by GSEA software (v4.1.0, https://www.gsea-msigdb.org/).

### 2.5. Immunity Analysis of the Signature

To uncover the immune activity of the RRlncRNAs signature, six immunity algorithms, including TIMER, CIBERSORT, QUANTISEQ, MCPCOUNTER, XCELL, and EPIC, were applied to evaluate immune responses between two risk subgroups by single-sample gene set enrichment analysis (ssGSEA) [16].

### 2.6. Competing Endogenous RNA (ceRNA) Network Construction

The DIANA prediction tools were used to detect the target miRNAs of each lncRNA and the threshold for the relationship between lncRNAs and miRNAs was set to 0.95. Then, we explored the potential target mRNAs binding to miRNAs using three miRNA databases (miRDB, TargetScan, and miRTarBase). Based on the predicted correlation of lncRNA-miRNA and miRNA-mRNA, the ceRNA network was created by Cytoscape.

### 2.7. Functional Enrichment Analysis of the Target mRNAs

To determine the underlying function of mRNAs in the ceRNA regulatory network, we conducted the Gene Ontology (GO) function and the Kyoto Encyclopedia of Genes and Genomes (KEGG) pathway enrichment analysis by “clusterProfiler” package. *p* < 0.05 was found statistically important.

### 2.8. Cell Culture and Cell Transfection

Two human NSCLC cell lines (A549 and NCI-H520) and one human lung epithelial cell line (BEAS-2B) were purchase from the Chinese Academy of Sciences (Shanghai, China). Above NSCLC cells and BEAS-2B were cultured in RPMI-1640 medium (KeyGEN, Nanjing, China) with 10% fetal bovine serum (FBS, PAN-Seratech, Germany) and 1% penicillin/streptomycin with 5% CO_2_ at 37°C. We purchased siRNA negative control and si-LINC01977 from Ribobio Biotechnology and transfected them into NSCLC cell lines using the Lipotransfectamine 3000 (Thermo). The target sequence of si-NC and si-LINC01977 are presented in Supplementary Table S1. After 48 h, transfected NSCLC cells were collected for the next *in vitro* experiments.

### 2.9. Quantitative Real-Time PCR (qRT-PCR)

Total RNA was extracted from cell lines via RNA-easy isolation reagent (Vazyme biotech) and was reversed into synthesizing complementary DNA (cDNA) using PrimeScript Mix reagent (Takara). The PCR reaction system was prepared to utilize SYBR Green® Premix Ex Taq™ (Vazyme biotech). Results of individual lncRNAs were normalized to the expression of GAPDH. The primer sequences for each gene are shown in Supplementary Table S2.

### 2.10. Clonogenic Survival Assay

NSCLC cells were seeded in 6-well plates at 200, 400, 800, 1600, and 3200 cells/well. 24 hours later, we irradiated them with a single dose of 0, 2, 4, 6, and 8 Gy, respectively. Then, cultured for 2 weeks later, 1% crystal violet was applied in staining clonogenic cells. The clonogenic survival curves were generated according to the following formula model: SF = 1 − (1 − *e*^−kd^)^*n*^ as previously described [17].

### 2.11. Immunofluorescence Assay

The immunofluorescence of *γ*H2AX was detected by the anti-*γ*H2AX primary antibody (1 : 500, Abcam, UK) and the detailed process was described in the previous protocol [17]. Cells were snapped under a confocal fluorescence microscope (Leica).

### 2.12. Statistical Analysis

R software (4.0.1) was utilized for all statistical analyses. Differences in OS of both risk groups were assessed through the Kaplan–Meier analysis. By the combination of univariate and multivariate Cox regression methods, the independence of our risk model was detected. In addition, receiver operating characteristic (ROC) analysis was utilized to examine the reliability of our risk model. *p* < 0.05 was considered statistically significant for each analysis.

## 3. Results

### 3.1. Determination of Radioresponse-Related lncRNAs

**Figure 1:**
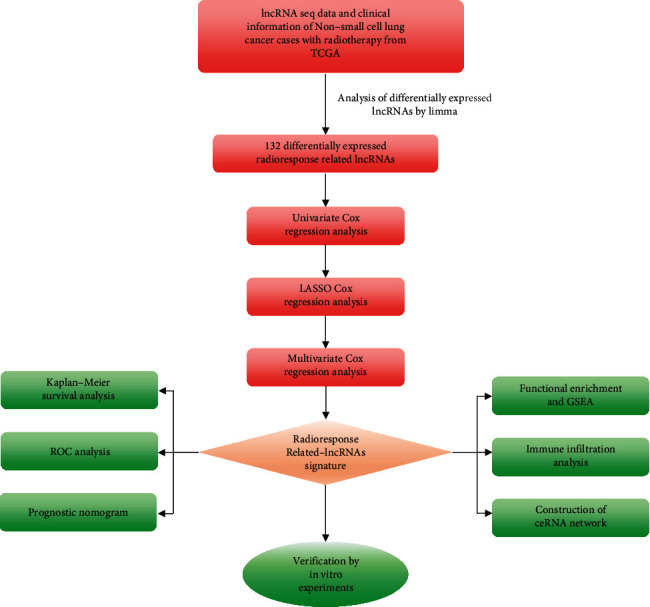
Brief flowchart of the whole study.

**Figure 2:**
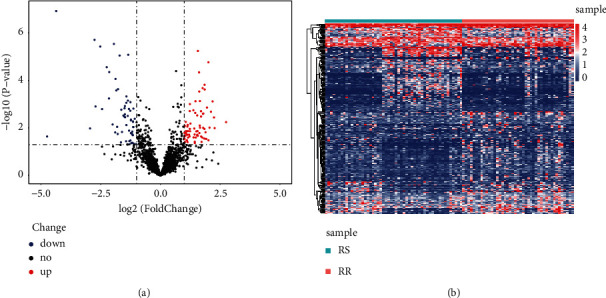
Determination of differentially expressed radioresponse-related lncRNAs (DERRlncRNAs). (a) Heat map of DERRlncRNAs in NSCLC patients with radiotherapy history. (b) Volcano plot of DERRlncRNAs in NSCLC patients with radiotherapy history.

The infographic flowchart of the present study is shown in Figure 1. We selected 96 NSCLC cases for the entire analysis. The lncRNAs expressions were detected between 43 RR cases and 53 RS cases. Next, 60 highly expressed lncRNAs and 72 lowly expressed lncRNAs were obtained. Figures 2(a) and 2(b) revealed that radioresponse-related lncRNAs (RRlncRNAs) differed dramatically between RS and RR groups.

### 3.2. Construction of the RRlncRNAs Signature

**Figure 3:**
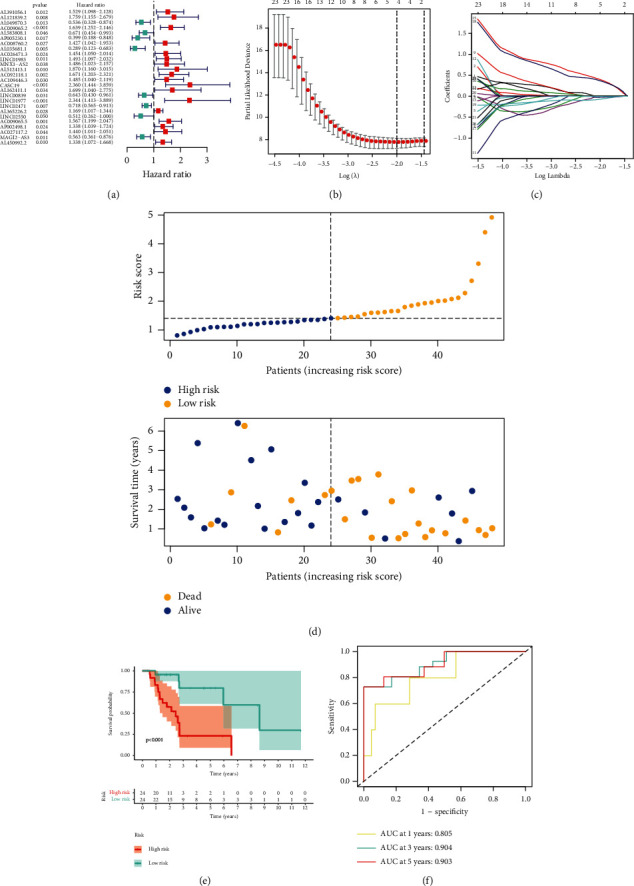
Identification of the radioresponse-associated lncRNAs biomarker model in the training cohort. (a) Univariate Cox analysis for determination of prognostic radioresponse-associated lncRNAs. (b) LASSO regression method for construction of radioresponse-related lncRNAs signature. (c) LASSO coefficient of 26 radioresponse-related lncRNAs in NSCLC. (d) Distribution of increasing risk scores based on radioresponse-related lncRNAs (upper) and clinical outcome of NSCLC cases with increasing risk scores (below). (e) Kaplan–Meier curves of NSCLC cases were classified by a median value of risk. (f) ROC methods reveal the potentiality of employing the risk score to forecast the prognosis of NSCLC patients.

Firstly, we randomly divided all samples into training cohort (*n* = 48) and test cohort (*n* = 48). After applying the univariate Cox method on the training set, we screened 26 representative prognostic lncRNAs significantly correlated with survival (Figure 3(a)). Then, LASSO-penalized regression was applied to diminish the overfitting of signature (Figures 3(b) and 3(c)). Moreover, a signature of four radioresponse-related lncRNAs (CASC19, LINC01977, LINC02471, and MAGI2-AS3) was built up by the multivariate Cox analysis (Table 2). The risk value of each NSCLC case was generated using the following formula: [exp CASC19 × (0.3999)] + [exp LINC01977 × (0.3693)] + [exp LINC02471 × (−0.0701)] + [exp MAGI2 − AS3 × (−0.0238)]. Subsequently, all patients were separated into the high-risk score group and low-risk score group by the median risk value. The layout of risk score and OS outcomes of NCSLS patients in two risk subgroups are presented in Figure 3(d).

**Figure 4:**
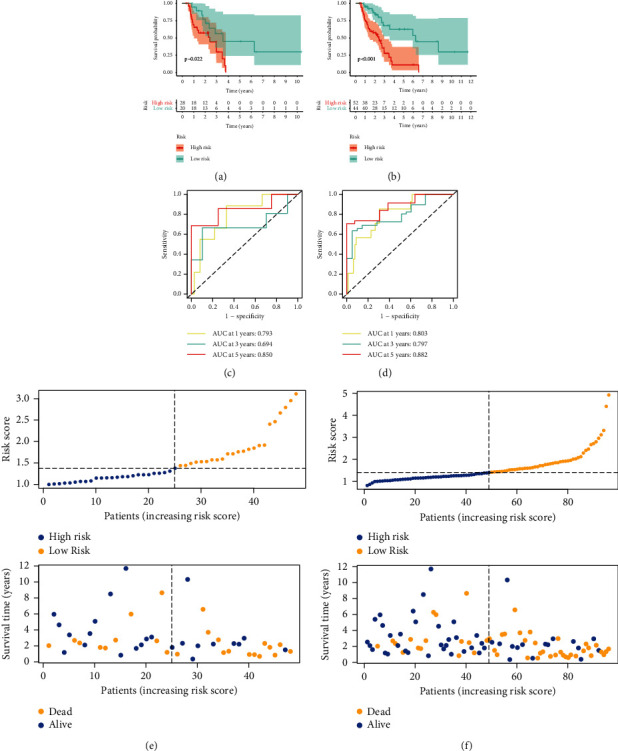
Verification of the radioresponse-associated lncRNAs biomarker model. Kaplan–Meier curves for both risk groups in the test set (a) and entire set (b). ROC curves for verifying model performance in the prediction of NSCLC prognosis in the test set (c) and entire set (d). Predictive characteristics of four radioresponse-related lncRNAs' biomarker model in the test set (e) and entire set (f).

In the training cohort, the KM survival curves indicated a notable difference in survival outcome between the low-risk group and high-risk group (Figure 3(e)). ROC plots were applied to measure the reliability of our constructed model. The area under the ROC (AUC) values of 1-, 3-, and 5-year OS rates were 0.805, 0.904, and 0.903, pointing out the powerful predictive ability of the signature (Figure 3(f)). Similarly, the survival outcome of cases could be distinguished well by the model through KM survival curves in both the test set and entire cohort (Figures 4(a) and 4(b)). Meanwhile, the value of AUC for the test cohort and entire set is shown in Figures 4(c) and 4(d). In addition, risk score layout and OS outcome in the test set and entire cohort are shown in Figures 4(e) and 4(f).

### 3.3. Development of the Radioresponse-Based Nomogram

**Figure 5:**
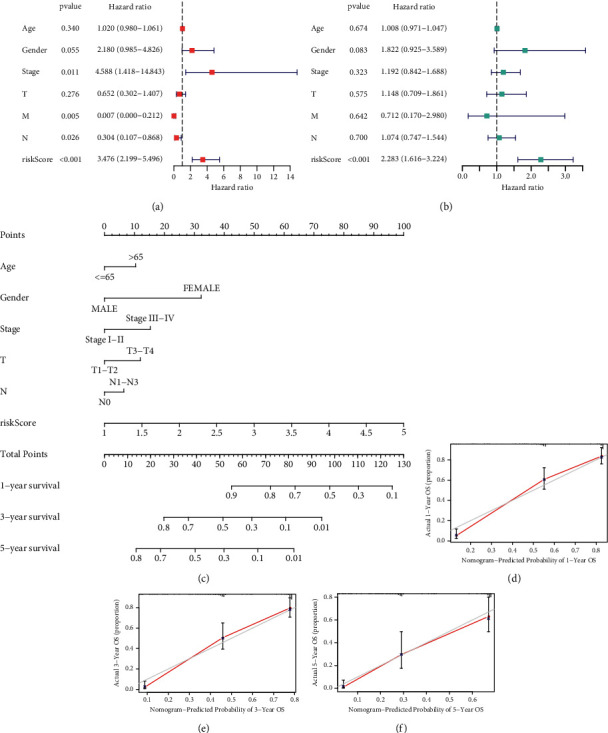
A nomogram based on radioresponse-related lncRNAs biomarker model and clinical parameters. The results of independent prognostic analysis by univariate Cox regression (a) and multivariate Cox regression (b). (c) A radiobiological nomogram created by combining signature and clinical parameters. (d-f) Calibration curves for the predictive probability and accuracy of the radiobiological nomogram.

To determine the independence of the four-RRlncRNA signature, we carried out univariate and multivariate Cox methods to analyze the clinical characteristics and risk scores generated by the model. As shown in Figure 5(a), stage (*p*=0.011), M stage (*p*=0.005), N stage (*p*=0.026), and risk score (*p* < 0.001) were meaningful for predicting survival outcome. Multivariate Cox analysis disclosed that only risk score (*p* < 0.001) was remarkably correlated with survival time, suggesting our signature could be independent of the clinical factors (Figure 5(b)). Afterward, a survival prognosis nomogram was established with clinical characteristics and a radioresponse-based model in the entire cohort (Figure 5(c)). At the same time, the calibration curves for the predictive ability of 1, 3, and 5 years presented no declination between the ideal reference line and prediction by prognostic nomogram (Figures 5(d)–5(f)).

### 3.4. GSEA Enrichment of the Signature

**Figure 6:**
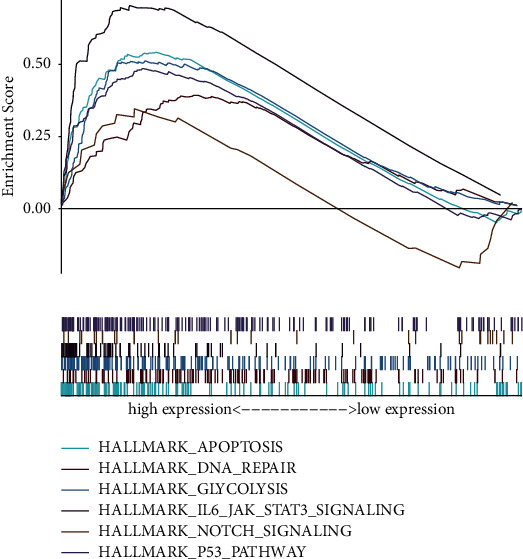
Gene set enrichment analysis (GSEA) of the two risk groups.

As displayed in Figure 6, the top six hallmark pathways were “p53 pathway,” “IL6/JAK/STAT3 pathway,” “Notch pathway,” “DNA repair,” “glycolysis,” and “apoptosis.” The results indicated that these hallmark pathways related to radioresistance regulation were activated in the patients with the high-risk group.

### 3.5. The Association between the Clinical Traits and the Signature in NSCLC

**Figure 7:**
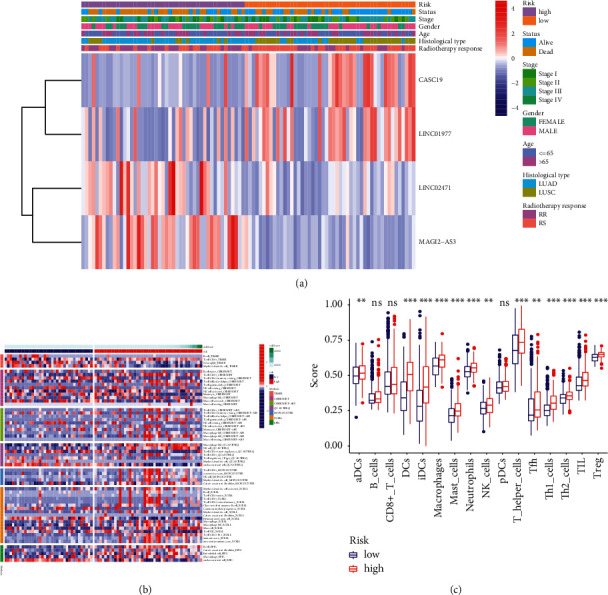
The association of the radiobiological model with different clinical factors and immune microenvironment. (a) Heat map of the radiobiological signature with different clinical traits. (b) Heat map for radiobiological model and immune activity. (c) ssGSEA for the relationship between 16 types of immunocyte subpopulations and risk score (^*∗*^*p* < 0.05; ^∗∗^*p* < 0.01; ^∗∗∗^*p* < 0.001).

To evaluate the clinical potency of our signature, we employed clinical relative analysis. The heat map demonstrated that the high-risk score was remarkably related to the status, stage, gender, age, histological type, and radiotherapy response (Figure 7(a)).

### 3.6. Immune Activity Analysis of the Signature

Immune microenvironment has been proved to be closely bound up with the regulation of tumor progression and radioresistance [18, 19]. To investigate the power of our model for mirroring the condition of the immune microenvironment in NSCLC, we performed immunity analysis by the ssGSEA method.  Figure 7(b) shows the difference in immune responses between both risk score groups. We then estimated the association between risk score and immunocyte subpopulations infiltration by ssGSEA of TCGA-NSCLC. As uncovered by (Figure 7(c)), except for B cells, CD8 T cells, and pCDs, the remaining immunocyte populations were highly enriched in the high-risk group.

### 3.7. Construction and Functional Analysis of the Radioresponse-Related ceRNA Network

**Figure 8:**
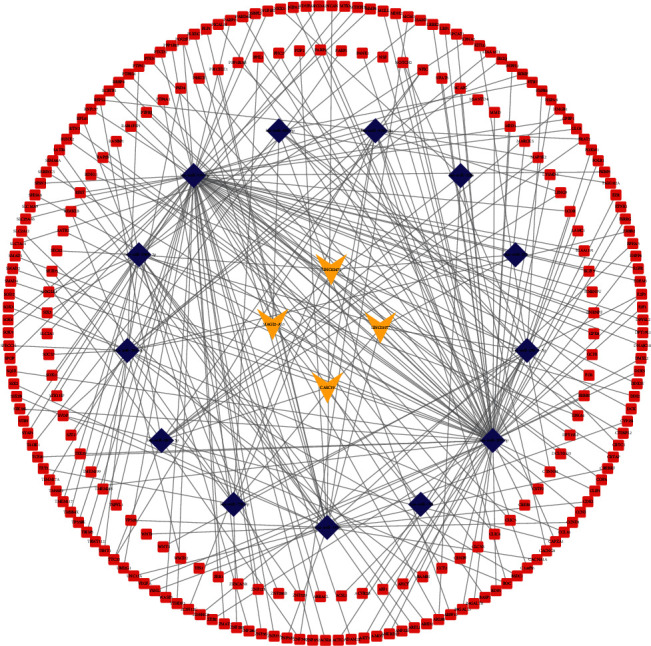
Creation of radiobiological signature-based ceRNA networks.

**Figure 9:**
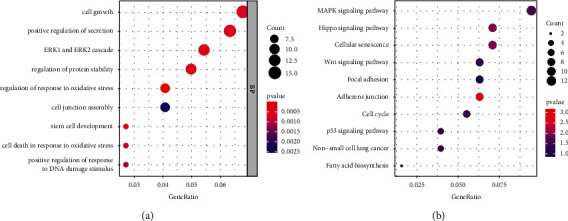
Functional annotation of mRNAs from radiobiological ceRNA networks. (a) GO biological analysis of mRNAs. (b) KEGG signaling pathway analysis of mRNAs.

Currently, a growing body of evidence indicates that lncRNAs could bind with microRNAs (miRNAs) to regulate the expression s of target mRNAs, which may modulate the biological process of malignant tumors [20–22]. We set up a ceRNA regulatory network consisting of four lncRNAs, 15 miRNAs, and 257 mRNAs based on interaction relationships predicted by DIAND, miRDB, TargetScan, and miRTarBase (Figure 8). Moreover, GO and KEGG analyses were implemented to better unearth the underlying function of the network.  Figure 9(a) illustrates that the most distinctively enriched biological process contained “cell growth,” “positive regulation of secretion,” “ERK1 and ERK2 cascade,” “response to oxidative stress,” “cell junction assembly,” and “DNA damage stimulus.” In terms of the KEGG pathway, the main important pathways included “MAPK pathway,” “Hippo pathway,” and “Wnt pathway” and “p53 pathway” (Figure 9(b)).

### 3.8. Determination of the Expression Patterns and the Prognostic Performance of the Four-lncRNA Signature

**Figure 10:**
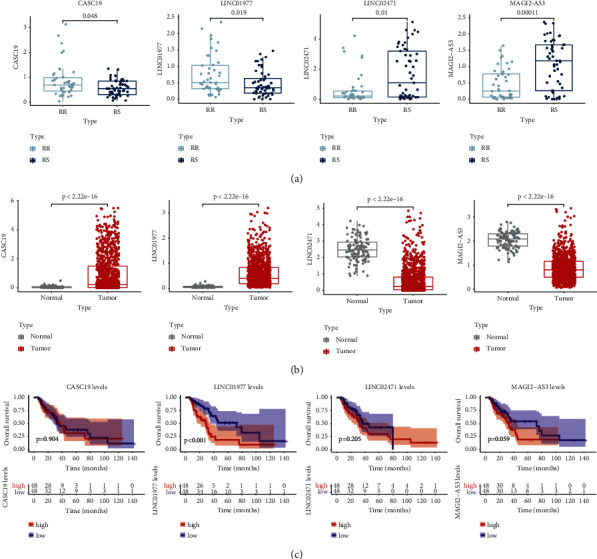
Expression pattern and survival prognosis of CASC19, LINC01977, LINC02471, and MAGI2-AS3. (a) The expression levels of CASC19, LINC01977, LINC02471, and MAGI2-AS3 in the radiosensitive (RS) group and radioresistant (RR) group. (b) The expression levels of CASC19, LINC01977, LINC02471, and MAGI2-AS3 in NSCLC and adjacent normal tissues. (c) Kaplan–Meier curves for the four signature lncRNAs.

As revealed by Figure 10(a), CASC19 and LINC01977 were greatly upregulated in NSCLC carcinoma than in normal tissues, whereas LINC02471 and MAGI2-AS3 were downregulated in normal tissues. In addition, CASC19 and LINC01977 were highly expressed in the RR group, while LINC02471 and MAGI2-AS3 had lower expressions in the RR group (Figure 10(b)). Based on the dataset of TCGA-NSCLC patients with radiotherapy history, KM survival analysis indicated that LINC01977 was accompanied with dismal OS in NSCLC patients with RT (Figure 10(c)).

### 3.9. Inhibition of LINC01977 Enhanced Radiosensitivity in NSCLC

**Figure 11:**
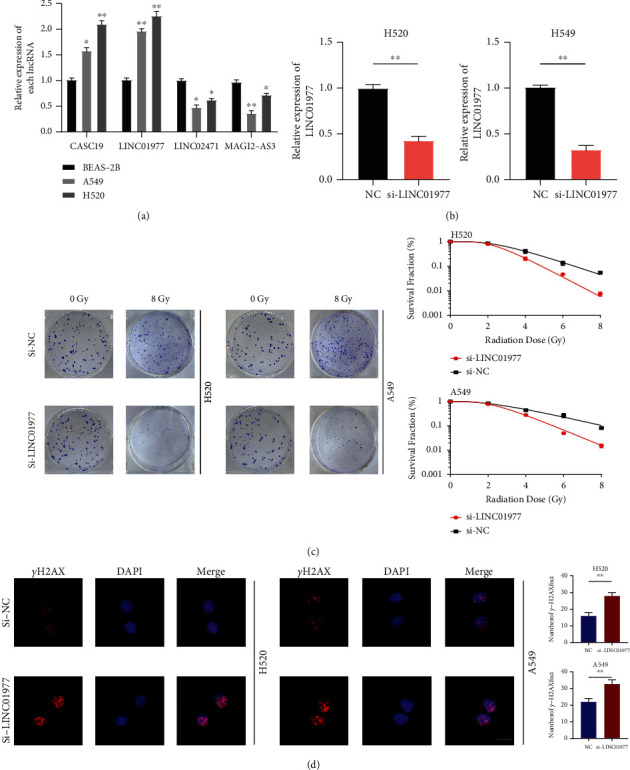
Inhibition of LINC01977 boosts radiosensitivity of NSCLC cells. (a) Detection of four signature lncRNAs' expression by qRT-PCR. (b) Transfection efficiency of inhibition LINC01977 by siRNA. (c) Clonogenic survival assay in H520 and A549. (d) LINC01977 knockdown boosted *γ*H2AX after irradiation in H520 and A549 (scale bar = 10 *μ*m; ^*∗*^*p* < 0.05; ^∗∗^*p* < 0.01; ^∗∗∗^*p* < 0.001).

Firstly, we applied the qRT-PCR assay to confirm the expression level of four lncRNAs in BEAS-2B, H520, and A549 (Figure 11(a)). Subsequently, we selected LINC01977 for further *in vitro* experiment. As observed from Figure 11(b), LINC01977 expression evidently downregulated within H520 and A549 cells by si-LINC01977 transfection. By performing a clonogenic survival assay, the roles of TRPM2-AS on the radiosensitivity of NSCLC cells were detected. After being exposed to an increasing dose of irradiation (IR), colony survival fractions were dramatically reduced by LINC01977 downregulation (Figure 11(c)). The same results were acquired by DNA damage assay. We found that NSCLC cells with si-LINC01977 greatly promoted the expression of *γ*H2AX (DNA damage marker) after receiving an 8 Gy dose of RT (Figure 11(d)).

## 4. Discussion

Non-small-cell lung cancer (NSCLC) is a primary thoracic tumor with a terrifically malignant behavior, which is the leading cause of cancerous death. Despite the considerable success of radiotherapy in the treatment of NSCLC, radioresistance is still considered a major obstacle to effective radiotherapy. Therefore, it is highly essential to unearth novel biomarkers predicting radiation response, to obtain new therapeutic targets and develop new treatment options. Accumulated evidence has reported that lncRNAs act as regulators in NSCLC radioresistance [23]. Recently, researchers have attached more attention to lncRNA-based signatures because of their powerful predictive ability compared to TNM staging systems or standard benchmarks [24–26]. However, prognostic markers based on radioresponse associated lncRNA expression profiling have not been analyzed in NSCLC cases with RT history.

Here, according to the TCGA-NSCLC project, we created a newly radioresponse-related lncRNAs signature that could accurately determine high-risk patients and exploit the clinical utility in NSCLC patients. We first collected 132 differentially expressed radioresponse-related lncRNAs based on 96 NSCLC patients who received RT. Then, four radioresponse-related lncRNAs were screened by Cox relative regression methods in the training set. These four lncRNAs were used to build up a risk model for forecasting the survival outcome of NSCLC samples. KM survival method demonstrated a notable difference in OS between the two risk subgroups. Both the test cohort and the entire cohort were employed to confirm the above results. To simplify our proposed model, we created a radiobiological nomogram by combining the RRlncRNAs signature and other clinical factors. The calibration plots indicate that our model exhibits a favorable fit and better clinical effectiveness.

In order to exploit the clinical potency of our radiobiological model, we analyzed the relationship between clinical traits and the signature and observed that low-risk score negatively associated with stage, age, and radiotherapy response. We further explored the lncRNA-related ceRNA network to elucidate the underlying biological function and potential pathway of the selected four lncRNAs. There were many radioresistance associated signal pathways about ceRNA network regulation, such as MAPK pathway,” “Hippo pathway,” and “Wnt pathway” and “p53 pathway”.

To explore the possible biological function of the signature, the GSEA method was employed. GSEA revealed that the gene sets in the high-risk score group were enriched in “DNA repair” “glycolysis,” “apoptosis,” and pathways related to cancer, including p53 pathway, JAK-STAT3 signal pathway, and Notch signal pathway, providing strong evidence that these Hallmark pathways play a central part in in the resistance to radiotherapy in NSCLC. The main mechanism of irradiation-induced tumor cell death is DNA damage. However, cancer cells could correct DNA double-strand breaks by activating DNA damage repair, subsequently promoting radioresistance and tumor cell survival [27]. Glucose uptake supplies energy and biosynthetic materials for tumor cell proliferation [28]. Overexpression of glucose transporter proteins and glycolytic enzymes are common oncogenic signals [29, 30]. Currently, several studies have shown that the radiosensitivity of NSCLC can be enhanced by modulating glycolysis [31]. It has been shown that there is a large amount of P53-dependent apoptosis in irradiation-sensitive tissues, but p53-deficient proved notable radioresistance in the mouse model [32, 33]. Targeting the P53 signaling pathway could enhance radiosensitivity, which has been demonstrated in a variety of cancers [34–36]. Notch signaling mainly regulates the cell cycle, inhibits apoptosis by suppressing PTEN expression, and triggers tumor progression together with PI3K-AKT signaling [37]. Activation of the Notch pathway in NSCLC patients is closely bound up with a worse prognosis, and cancer cells with high Notch expression are more resistant to irradiation [38].

Radiotherapy-induced immunosuppressive changes in the TME are conducive to the immune escape of cancer cells, which in turn confers radioresistance in tumors [18]. Thus, we evaluate the condition of the NSCLC immune microenvironment by the ssGSEA algorithm. As revealed by immunocyte infiltration analysis, patients with high risk showed dramatically higher proportions of M2-like macrophages. M2-like macrophage, as a protumor subtype of tumor-associated macrophage (TAM), shows radioresistance activity in TME. Apoptotic cells, appearing after radiotherapy, activate macrophages with the M2 phenotype to secrete a range of cytokines such as TGF-B which could induce radioresistance and block the development of ”tumor vaccine” [39–41]. Zhang et al. revealed that M2 macrophage-derived exosome could heighten resistance to RT through transferring AGAP2-AS1 into lung cancer cells [42]. Other immunosuppressive immune Tregs were also highly infiltrated in a high-risk group. The existence of Tregs might curb the effectiveness of RT. With the increased recruitment level of Tregs after irradiation, the immunomodulatory effects induced by RT were greatly reduced [43]. In addition, Treg is more resistant to irradiation than other T cell populations due to its increased expression of Akt [44].

In the present study, our constructed risk signature consisted of four lncRNAs which were closely related to NSCLC prognosis. Among these five lncRNAs, CASC19 and LINC01977 are potential risky indicators, but LINC02471 and MAGI2-AS3 are potential protective indicators. CASC19 (Cancer Susceptibility 19) has been reported to mediate the progression and radioresistance of several tumors. In pancreatic cancer (PC), CASC19 could facilitate malignant growth and metastasis of PC via miR-148b/E2F7 ceRNA axis with sponge activity [45]. As suggested by Liu et al., CASC19 was highly expressed in radioresistant nasopharyngeal carcinoma (NPC) cells and could confer radioresistance in NPC through autophagy-related AMPK-mTOR pathway [46]. In addition, CASC19 also has an oncogenic effect on NSCLC. Wang et al. found that CASC19 could bind with miR-301b-3p to regulate the expression of low-density lipoprotein receptor (LDLR), which promotes NSCLC cell proliferation and metastasis [47]. The LINC01977 is a novel carcinogenic promoter reported in breast cancer (BC). Silencing LINC01977 could greatly repress BC development and chemoresistance to doxorubicin. In terms of molecular mechanism, LINC01977 might sponge miR-212-3p to attenuate the expression of the GOLM1 gene [48]. Notably, LINC01977 has not been studied in lung cancer, which suggests that we can do further investigation. LINC02471, a member of the intergenic lncRNA family, is recognized as a crucial enhancer in papillary thyroid carcinoma (PTC). Chen et al. observed that downregulation of LINC02471 could hinder PTC cell metastasis and trigger l apoptosis by binding with miR-375, indicating that it can be considered as a potent predictor for metastasis of patients with PTC [49]. MAGI2-AS3 was originally identified as an antisense RNA of Membrane Associated Guanylate Kinase 2 (MAGI2) involved in regulating the malignant behaviors of various types of cancers. As discovered by Cheng et al., MAGI2-AS3 could enhance sensitivity to radiotherapy in esophageal cancer by inhibiting the HOXB7 gene, offering a valuable molecular marker for radioresistance [50]. Another research studied that bladder cancer (BC) patients with upregulation of MAGI2-AS3 display a lower incidence of cancer metastasis. MAGI2-AS3 may reinforce the stability of MAGI2 to mediate epithelial-mesenchymal transition (EMT) of BC [51]. Our results are in line with these studies, suggesting the MAGI2-AS3 is a protective factor (HR < 1) in NSCLC.

Finally, we explored the relationship between LINC01977 expression and radioresistance in NSCLC cell lines. We found that silencing LINC01977 significantly suppressed clonogenic survival and boosted DNA damage repair, implying that LINC01977 could be a possible therapeutic target for promoting radiotherapy sensitivity in NSCLC patients.

There are some limitations to our study. First, all research populations processed in this subject were merely collected from the TCGA database. The large-scale clinical cohort data or other external datasets need to be warranted to confirm our signature. Moreover, considering our in vitro assays mainly focus on the cell phenotype of tumor, molecular mechanism and in vivo experiments are need to verify the results in further exploration.

In summary, we firstly created a radioresponse-related lncRNAs signature based on four prognostic lncRNAs associated with radiation therapy which offer a reliable reference for the prognostic forecast. Our subject brings new sights into the clinical strategy of NSCLC patients who received radiotherapy.

## Figures and Tables

**Table 1:** Clinicopathologic characteristics of NSCLC patients with radiotherapy.

Features	Radioresistance group	Radiosensitive group
Total	43 (100%)	53 (100%)

*Age*		
＞65	19 (44.2%)	23 (43.4%)
≤65	24 (55.8%)	30 (53.6%)

*Gender*		
Male	23 (53.5%)	31 (58.5%)
Female	20 (46.5%)	22 (41.5%)

*Stage*		
Stage I	8 (18.6%)	15 (28.3%)
Stage II	9 (20.9%)	14 (26.4%)
Stage III	22 (51.2%)	22 (41.5%)
Stage IV	4 (9.3%)	2 (3.8%)

*T* s*tage*		
T1	8 (18.6%)	11 (20.7%)
T2	23 (53.5%)	31 (58.5%)
T3	10 (23.2%)	8 (15.1%)
T4	1 (2.33%)	3 (5.7%)
Unknown	1 (2.33%)	

*N stage*		
N0	15 (34.9%)	19 (35.8%)
N1	11 (25.6%)	12 (22.6%)
N2	15 (34.9%)	18 (34.0%)
N3	1 (2.3%)	2 (3.8%)
Unknown	1 (2.3%)	2 (3.8%)

*M stage*		
M0	29 (67.4%)	43 (81.1%)
M1	4 (9.3%)	2 (3.8%)
Unknown	10 (23.3%)	8 (15.1%)

**Table 2:** Four radioresistance-related lncRNAs remarkably correlated with OS.

lncRNA	Coefficient	Hazard ratio (95% CI)	*P* value
CASC19	0.3999	2.36 (1.44–3.86)	<0.001
LINC01977	0.3698	2.34 (1.41–3.89)	0.012
LINC02471	−0.0702	0.72 (0.57–0.91)	0.027
MAGI2-AS3	−0.0238	1.53 (1.20–1.95)	<0.001

## Data Availability

The public datasets to support the results of this subject can be gained from TCGA (https://portal.gdc.cancer.gov/).

## Supplementary Materials

Supplementary Materials Supplementary Table 1: target sequence of si-NC and si-LINC01977. Supplementary Table 2: primers utilized for qRT-PCR.
