# Supplementary material for: A Radioresponse-Related lncRNA Biomarker Signature for Risk Classification and Prognosis Prediction in Non-Small-Cell Lung Cancer
Source: J Oncol. 2021 Sep 21;2021:4338838. doi: 10.1155/2021/4338838 (PMC8478572; doi:10.1155/2021/4338838)
Supplement: Supplementary Materials — Supplementary Table 1: target sequence of si-NC and si-LINC01977. Supplementary Table 2: primers utilized for qRT-PCR. [file 4338838.f1.zip › 4338838.f1/Supplementary Table 1.pdf]

Supplementary Table 1. Target sequence of si-NC and si-LINC01977

Gene	Target sequence
si-NC	AATTCACTCCAAGTCTCTTCC
si-LINC01977	TGTTCCTAATTTGGACACTGGTTTA
