# Supplementary material for: A Radioresponse-Related lncRNA Biomarker Signature for Risk Classification and Prognosis Prediction in Non-Small-Cell Lung Cancer
Source: J Oncol. 2021 Sep 21;2021:4338838. doi: 10.1155/2021/4338838 (PMC8478572; doi:10.1155/2021/4338838)
Supplement: Supplementary Materials — Supplementary Table 1: target sequence of si-NC and si-LINC01977. Supplementary Table 2: primers utilized for qRT-PCR. [file 4338838.f1.zip › 4338838.f1/Supplementary Table 2.pdf]

Supplementary Table 2. Primers utilized for qRT-PCR

Gene	5'to 3'
CASC19	Forward TTTAGCCTGCATAGGACCCTC<br>Reverse GTCTGGTCAAATTACAATCAGTTGG
LINC01977	Forward GGACACTGGTTTACGAAAGT<br>Reverse AATGACACGGCTCTACGC
LINC02471	Forward ATGCTAAACACTGCCTCATCTCT<br>Reverse GATTAGCTGCTTCCCAGTGT
MAGI2-AS3	Forward CCAGTGCGGACCTTTCTTCA<br>Reverse CTCTTGGATGCAAACGGCAG
GAPDH	Forward TGCACCACCAACTGCTTAGC<br>Reverse GGCATGGACTGTGGTCATGAG
